# Comparison of distance versus in-person laparoscopy training using a low-cost laparoscopy simulator—a randomized controlled multi-center trial

**DOI:** 10.1007/s00464-024-11069-2

**Published:** 2024-09-13

**Authors:** Mark Enrik Geissler, Jean-Paul Bereuter, Rona Berit Geissler, Guus Mattheus Johannes Bökkerink, Luisa Egen, Karl-Friedrich Kowalewski, Caelan Haney

**Affiliations:** 1 Else Kroener Fresenius Center for Digital Health, Faculty of Medicine and University Hospital Carl Gustav Carus, TUD Dresden University of Technology, 01307 Dresden, Germany; 2 Department of Urology and Urosurgery, University Medical Centre Mannheim, University of Heidelberg, Mannheim, Germany; 3 Princess Máxima Center for Pediatric Oncology, Princess Maxima Center, Utrecht, The Netherlands; 4 Division Intelligent Systems and Robotics in Urology, German Cancer Research Center (DKFZ), Heidelberg, Germany; 5 DKFZ Hector Cancer Institute at the University Medical Center, Mannheim, Germany

**Keywords:** Minimally invasive surgery, Laparoscopic skill analysis, Laparoscopy, Simulation training

## Abstract

**Introduction:**

Simulation training programs are essential for novice surgeons to acquire basic experience to master laparoscopic skills. However, current state-of-the-art laparoscopy simulators are still expensive, limiting the accessibility to practical training lessons. Furthermore, training is time intensive and requires extensive spatial capacity, limiting its availability to surgeons. New laparoscopic simulators offer a cost-effective alternative, which can be used to train in a digital environment, allowing flexible, digital and personalized laparoscopic training. This study investigates if training on low-cost simulators in a digital environment is comparable to in-person training formats.

**Materials and methods:**

From June 2023 to December 2023, 40 laparoscopic novices participated in this multi-center, prospective randomized controlled trial. All participants were randomized to either the ‟distance” (intervention) or the “in-person” (control) group. They were trained in a standardized laparoscopic training curriculum to reach a predefined level of proficiency. After completing the curriculum, participants performed four different laparoscopic tasks on the ForceSense system. Primary endpoints were overall task errors, the overall time for completion of the tasks, and force parameters.

**Results:**

In total, 40 laparoscopic novices completed digital or in-person training. Digital training showed no significant differences in developing basic laparoscopic skills compared to in-person training. There were no significant differences in median overall errors between both training groups for all exercises combined (intervention 3 vs. control 4; *p* value = 0.74). In contrast, the overall task completion time was significantly lower for the group trained digitally (intervention 827.92 s vs. control 993.42; *p* value = 0.015). The applied forces during the final assessment showed no significant differences between both groups for all exercises. Overall, over 90% of the participants rated the training as good or very good.

**Conclusion:**

Our study shows that students that underwent digital laparoscopic training completed tasks with a similar number of errors but in a shorter time than students that underwent in-person training. Nevertheless, the best strategies to implement such digital training options need to be evaluated further to support surgeons’ personal preferences and expectations.

**Supplementary Information:**

The online version contains supplementary material available at 10.1007/s00464-024-11069-2.

Structured surgical training has proven vital to surgical novices, residents, and experienced surgeons to acquire and retain basic and advanced skills in minimally invasive surgery (MIS) among all operative fields [1, 2]. Simulation training has shown to benefit not only surgical skill development but also to improve patient outcomes [3]. However, even though training is an effective tool to develop surgical skills outside the operating room, the implementation into residency and uptake by surgeons varies broadly [4]. The reasons for this are manifold. For example, time and financial constraints associated with simulator-based laparoscopy training hinder the implementation of structured training [5, 6]. In addition, simulators require spatial capacity for storage and training. Training rooms are mainly located in the hospital, making it inflexible for trainees. Especially during prolonged times of absence from the hospital, simulator training is not possible. This might, for example, happen during pandemics, pregnancy or research stays abroad. Furthermore, young physicians often lack the time to perform additional simulator-based training after finishing clinical duties [7]. Recent studies consolidated the lack of structured surgical simulation training [8]. Therefore, new training possibilities are needed, reducing resource spending and allowing the training to be adapted to surgeons’ needs.

Throughout recent years, the already existing amount of available simulation systems, especially for laparoscopy and of low-cost systems, has increased substantially [9]. Low-cost trainers improve the accessibility to surgical training and reduce the financial burden on surgeons and institutions [10]. The “Laparoscopy Boxx-Pro” is such an evaluated low-cost surgical simulator [11]. Its easy set-up and high transportability allows it to be used as a simulator in any desired location. This opens up the opportunity for personalized at-home training in a digital manner [12]. Digital training does not require in-hospital spatial capacity and makes it more feasible for trainees to participate. The acquisition and development of basic laparoscopic skills via digital training have been shown to be feasible for surgical trainees and surgeons during an at-home laparoscopic training [13].

Digital surgical training has also proven to be sufficient to retain previously learned skills [14]. This is especially important in regard to more prolonged absences from the operating room as described previously. Digital training therefore shows great promise to counteract the loss of skills and the support of continuous skill development of laparoscopic surgical skills.

Nevertheless, the literature lacks studies comparing digital and in-person training in the foundational training of novice surgeons. This study aimed to compare the digital and in-person training using low-cost laparoscopy simulators for skill acquisition in a randomized fashion. Additionally, the aim was to test a structured curriculum and its applicability to digital training.

## Methods and analysis

This multi-center, prospective randomized, controlled, open-label study was approved by the ethics committee of the Technische Universität Dresden (EK 285072016). Participants were informed about the study and gave informed written consent to participate. This study was registered with the German trial registry (DRKS00034203) and written in accordance with the CONSORT (Consolidated Standards of Reporting Trials) statement [15].

### Participants

Medical students of all study years of the medical faculty of the University of Dresden and the Mannheim medical faculty of the University of Heidelberg were included as participants. All students participated voluntarily and provided informed consent to study participation and data analysis. Previous participation in minimally invasive surgery training courses was an exclusion criterion. Participants’ characteristics are shown in Table 1.

**Table 1:** Characterization of both training groups

Variable	In-person (*n* = 21)	Digital (*n* = 19)
Gender, [*n* (%)]		
Male	8 (38.1)	5 (26.3)
Female	13 (61.9)	14 (73.7)
Age, [years (SD)]	24.3 (3.1)	24.4 (3.1)
Year of study, [*n* (%)]		
1	3 (14.3)	6 (31.6)
2	0 (0)	0 (0)
3	2 (9.5)	1 (5.3
4	11 (52.4)	5 (26.3)
5	5 (23.8)	7 (36.8)
Experience in MIS, [*n* (%)]	3 (14.3)	4 (21.1)
Dominant hand, [*n* (%)]		
Right	20 (95.2)	18 (94.7)
Left	0 (0)	1 (5.3)
Both	1 (4.8)	0 (0)
Frequency of digital platform usage, [*n* (%)]		
Never	1 (4.8)	0 (0)
Daily	13 (61.9)	10 (52.6)
Weekly	6 (28.6)	8 (42.1)
Monthly	1 (4.8)	1 (5.3)
Participation in digital courses teaching practical skills, [*n* (%)]	2 (9.5)	6 (31.6)
Playing video games, [*n* (%)]	8 (38.1)	6 (31.6)
Visible loose knot	0 (0)	1 (5.3)
Balloon resection, [*n* (%)]		
No perforation	10 (47.6)	8 (42.1)
Micro-perforation	4 (19)	5 (26.3)
Macro-perforation	7 (33.3)	6 (31.6)

### Setting

Students randomized to the intervention group were trained in a digital manner, consisting of the regular training sessions taught online via a video conference platform. Participants’ exercises were monitored by the tutor. The place of training was free of choice to the students. The only limitation was a stable internet connection and the ability to follow the tutor’s instructions. Students randomized to the control group performed their training course in-person at the University of Dresden or Heidelberg/Mannheim. The rooms were blocked for training courses and 4–8 students trained in one training session under the tutor’s direct supervision.

### Randomization

The participants were randomized into two groups, resulting in one ‟digital” (intervention, *n* = 19) and one “in-person” (control, *n* = 21) group. Every participant was randomized using a research randomizer [16]. A random number was assigned to each member of both study groups.

### Training curriculum

The standardized training curriculum was supervised by experienced tutors of the surgical department of the University Hospital Carl Gustav Carus in Dresden. The curriculum was adapted to the Fundamentals of Laparoscopic surgery (FLS) and contained four basic laparoscopic tasks: Peg transfer, circle cutting, suture and knot, and Balloon resection extraction and has been described in previous studies [17, 18]. Each group participated in six training lessons supervised by experienced surgeons. At the beginning of each training students learned about the use of laparoscopic surgery and how to handle the instruments and two-dimensional view. Students started with rather easy exercises as the PEG transfer and surgical cutting and continued with more advanced tasks such as the resection and suture and knot task. The first two training sessions were used to teach the PEG and cutting exercise, followed by two sessions for the resection and suturing and knot task. During the last two sessions, participants were allowed to train all exercises until reaching proficiency and self-assessed their proficiency. The training was conducted over the course of two weeks with 3 training sessions each week and the final assessment in the following week. Tutors provided individual feedback during the trainers and intervened if problems occurred. Students were trained during the course until reaching a minimal proficiency level defined by Bechtolsheim et al. [18].

### Simulator

**Fig. 1:**
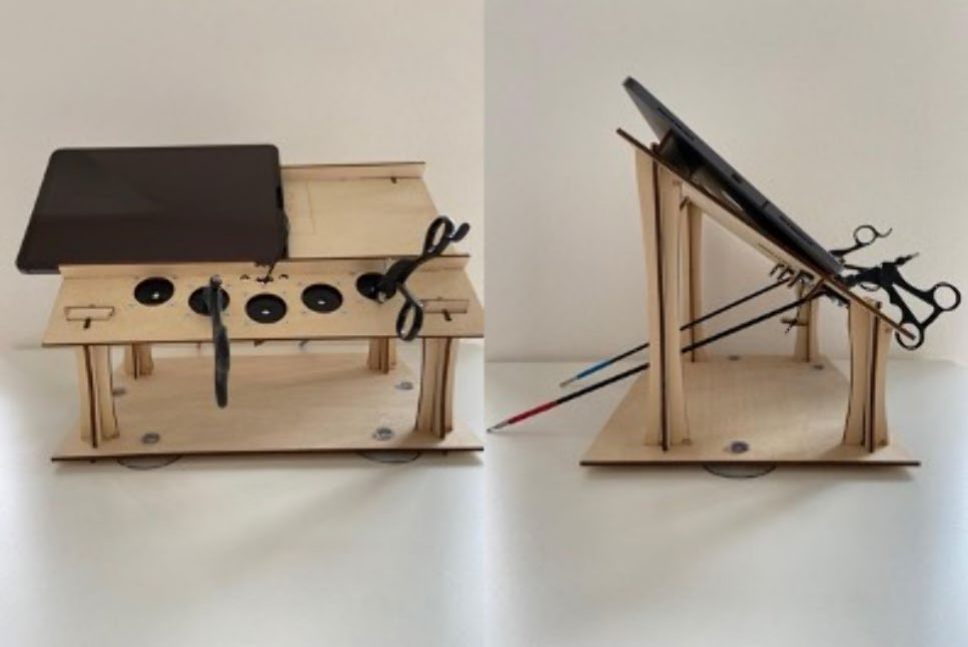
Overview of the used Laparoscopy Boxx simulator system

The simulator used for training purposes was the “Laparoscopy Boxx simulator” produced by the company Laparoscopy Boxx (Laparoscopyboxx, 2024; Fig. 1). The simulator costs 109 euro at that time and included basic training materials as well as the option to include 3 laparoscopic instruments for an additional 200 euro. The simulator is made out of wood and, through a click system, allows for easy set-up and usability (Fig. 1). The simulator is built to use a common smartphone or tablet as the camera system. Therefore, no large screen or expensive camera system is needed for use. The simulator is easy to disassemble and transport to a different place. During training, all students were questioned to bring a tablet and if not available this was provided by the study team.

### Intervention

The previously described training curriculum was performed by the control and intervention group. The intervention group received the simulators and required materials on the day of randomization. The participants were then trained at home using the video conference tool Zoom (Zoom Video Communications, Inc., 2024). Each online training session was conducted by two experienced trainers and a maximum of 20 study participants. The control group trained in-person at either the University of Dresden or the Medical University Mannheim/Heidelberg. The training was performed by two tutors with experience in laparoscopic surgery and training. The duration of online and in-person training lessons was one and a half hours. With six sessions, the complete training time was nine hours.

### Proficiency examination

**Fig. 2:**
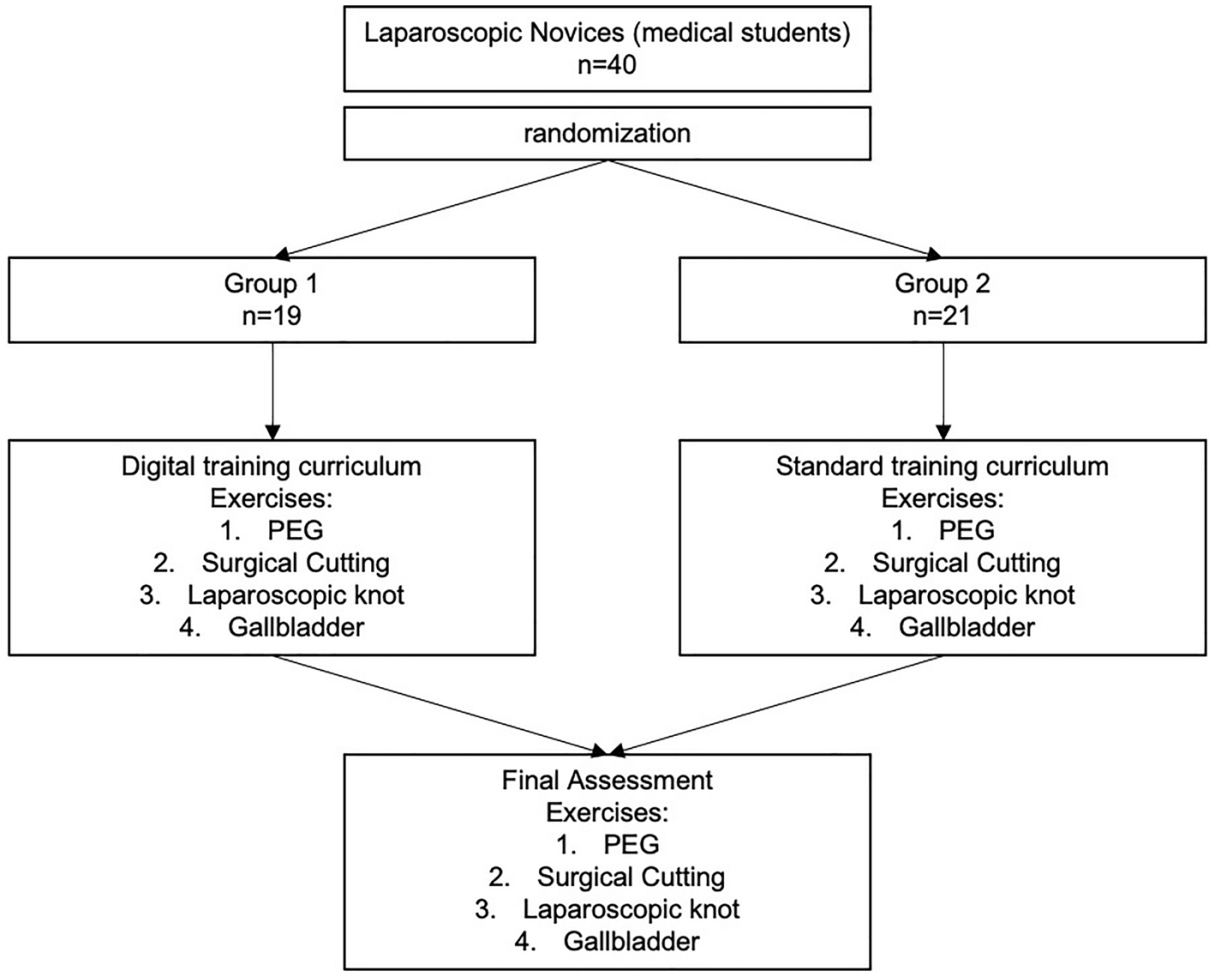
Flowchart showing an overview of the study process

The proficiency examination consisted of four exercises: Peg transfer, circle cutting, surgical suture and knot tying, and balloon resection (Fig. 2). The test was conducted using the ForceSense system (MediShield BV). All students were examined in the training facilities of the university clinic Dresden or Mannheim, using the same assessment set-up. The errors of each exercise were assessed as described in previous studies (Supplementary Table 1) [19]. Each participant was asked to answer a questionnaire NASA-TLX score and after scenario questionnaire (ASQ) [20, 21].

### Primary endpoints

#### Surgical errors

The assessment of errors was previously established and validated and can be seen in Supplementary Table 1. Errors were previously defined and assessed for each exercise. The errors include loss of Peg, cutting outside the line, and perforating the balloon. For the surgical knot and situation task, additional measures were used. The error score included insertion points of the needle, tightness of the knot, and adaptation of the vessel dummy (Penrose).

##### Overall time to task completion

The time for completion of each individual task was taken and these times were added together to form the overall task time measured in seconds.

##### Applied force

The applied force were evaluated for performance using the ForceSense system (MediShield BV). The laparoscopic simulation tool is designed to record the surgical field and motion of the laparoscopic instruments. Through a sensor below the mounted exercise tool, the measurement of force is possible. The applied force was further differentiated into mean non-zero force and maximal force during the course of the exercise. These parameters were already defined in a study conducted by Hardon et al. [22]:

- **Maximal force** in Newton [N]: The highest absolute force exerted by the instruments on the task platform during the task.
- **Mean non-zero force** [N]: Mean absolute force exerted by the instruments on the task platform during periods when force is not zero.
- **Task completion time** in seconds [s]: Time measured from the beginning of the task until task completion.

Lower results in mean, maximum force and task completion time indicate better performance.

#### Secondary endpoint

The ForceSense system (MediShield BV) allows for additional measurements, such as mean speed, volume of motion, and path length, which were measured during each exercise. The measured parameters were defined following previous studies [22, 23]:

- **Mean speed** [cm/s]: Average instrument speed per second.
- **Path length** [mm]: Total distance traveled by the tip of the right and left instrument together during the task.
- **Motion volume** [mm^3^]: Volume calculated, respectively, by the widest motion of the instruments of interest on the x-, y-, and z-axes.

Lower results in mean speed, path length, and motion volume indicate better results.

##### Task errors

The number of errors for each individual task was measured with lower errors indicating better performance.

##### Task time

The time for completion of each task was measured in seconds with less time needed for task completion indicating better results.

#### Cognitive workload and satisfaction with performance

For secondary outcome measures, participants’ subjective cognitive workload and satisfaction with task completion were analyzed using different questionnaires. Psychological parameters, such as mental demand or frustration, shown to be useful in surgical training, are determined using the NASA-TLX questionnaire [20]. This questionnaire is designed to assess participants’ cognitive workload during task performance. The questionnaire assesses mental, physical, and temporal demands, as well as performance, effort, and frustration levels. The score is performed after each exercise. To access the individual personal perception of performance the ASQ was also filled out after every exercise [21]. This questionnaire represents participants’ perceived satisfaction with the performance of the task. For the assessment of students demographic data a self-designed questionnaire was provided before the measurement. To also allow for the analyses of students’ perception regarding the different training regiments, a self-designed post-test questionnaire was distributed.

### Statistical analysis

The statistical analysis was conducted using SPSS version 28 (IMB Corp, Armonk NY, USA). In order to test data normality, the Kolmogorov–Smirnov test was used for continuous data and frequency distributions were applied where feasible. For general participant characteristics, either mean values were displayed with the corresponding standard deviations (SD) for continuous variables or relative and absolute frequencies used for categorical variables. To compare between groups, the appropriate tests (Mann–Whitney *U* test, Wilcoxon rank sum exact test, Welch Two-Sample *t* test, unpaired student’s *t* test) were used. Significance was defined as a *p* value equal to or smaller than 0.05.

### Sample size

The sample size was performed based on results of a previously performed similar study [19]. Assuming a two-sided significance level of 0.05 and a power of 0.8, a sample size of 17 participants per group would have been needed to detect a difference of mean errors of one. To account for possible dropouts, this number was rounded to 20 participants per group.

## Results

### Demographics and group characterization

In total, 40 participants were included in this randomized, open-label study. All students performed the course and reached proficiency. Students were randomly assigned to either the control (*n* = 21) or the intervention group (*n* = 19). The groups were evenly matched regarding demographic characteristics.

The majority of students used digital platforms on a daily or at least weekly basis, irrespective of training group (control: 19 vs. intervention: 18, *p* value = 0.664). A minority of students had experience of minimally invasive surgery in the operating room (control: 3 vs. intervention: 4, *p* value = 0.689). Nevertheless, no participating student had taken part in a previous minimally invasive surgery course. Further information about group characterization is shown in Table 1.

### Primary outcomes

#### Overall task errors

**Fig. 3:**
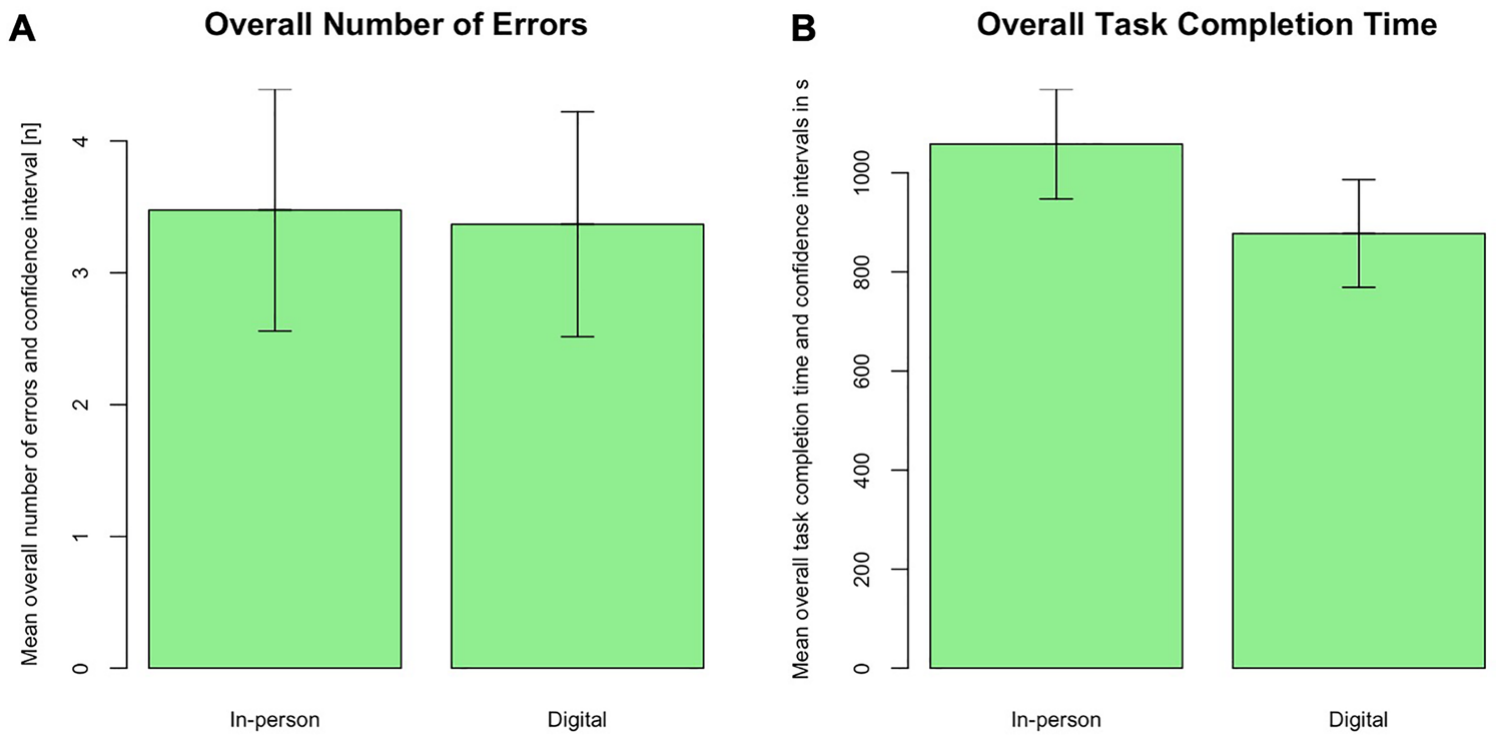
Overall number of errors and overall task completion time in seconds for the in-person and the digital training groups. **A** Comparison of the overall number of errors between both groups. **B** Comparison of the overall task completion time between both groups. Asterisk indicates a *p* value ≤ 0.05 between both groups

Overall, there were no significant differences for the mean occurrence of errors between both groups (control: 3.48 vs. intervention: 3.37, *p* value = 0.86) (Fig. 3A). The majority of students in both groups performed the Peg transfer task without any errors (control: 66.7% vs. intervention: 73.7%) and only a minority dropped Pegs more than once (control: 33.4% vs. intervention: 26.4%, *p* value = 0.056). Similarly, during the circle cutting task most students stayed within the boundaries (control: 71.4% vs. intervention: 68.4%) and only 6 students in the control group and 6 in the intervention group cut outside the boundaries (control: 28.5% vs. 31.6%, *p* value = 0.502). Nevertheless, during the suturing task, students struggled with precision. Most students missed either one or two stitching marks with no difference between both groups (control: 95.2% vs. intervention: 100%, *p* value = 0.062). The balloon resection was performed without perforation in nearly half of the cases (control: 47.6% vs. intervention: 42.1%) with several students’ performance resulting in micro- (control: 19% vs. intervention: 26.3%) or even macro-perforation (control: 33.3% vs. intervention: 31.6%) (*p* value = 0.856) (Table 2).

**Table 2:** Comparison of errors during the final test between the in-person and the digital training groups

Outcome parameter	In-person (*n* = 21)	Digital (*n* = 19)	*p* value
Mean overall errors (mean, SD)	3.48 (2.02)	3.37 (1.77)	0.86
Peg transfer number of dropped Pegs, [n (%)]			0.563
0	14 (66.7)	14 (73.7)	
1	3 (14.3)	4 (21.1)	
2	3 (14.3)	1 (5.3)	
3	1 (4.8)	0 (00	
Circle cutting, incorrect cuts [*n* (%)]			0.502
0–5 mm	15 (71.4)	13 (68.4)	
> 5 mm	2 (9.5)	4 (21.1)	
> 10 mm	4 (19)	2 (10.5)	
Suture and knot, [*n* (%)]			
Precise stitches			0.62
All correct	1 (4.8)	0 (0)	
One incorrect stitch	10 (47.6)	9 (47.6)	
Two incorrect stitches	10 (47.6)	10 (52.6)	
Penrose adaption			0.461
Completely adapted	18 (85.7)	14 (73.7)	
Partly adapted	3 (14.3)	4 (21.1)	
Not adapted	0 (0)	1 (5.3)	
Knot tightness			0.475
Tight knots	21 (100)	18 (94.7)	
Loose knot under pressure	0 (0)	0 (0)	
Visible loose knot	0 (0)	1 (5.3)	
Balloon resection, [n (%)]			0.856
No perforation	10 (47.6)	8 (42.1)	
Micro-perforation	4 (19)	5 (26.3)	
Macro-perforation	7 (33.3)	6 (31.6)	

Significant *p* values are highlighted in bold

#### Overall task completion time

The overall task completion time was significantly different between the in-person and the digital training group (control: 1058.1 s vs. intervention: 877.5 s, *p* value = 0.02) (Fig. 3B). When analyzing each task independently, the task completion time only differed significantly for the suture and knot task (control: 419 s vs. intervention: 320 s; *p* value = 0.015) between both measurements. This significant difference was not observed for the completion time of the Peg transfer (control: 145 s vs. intervention: 135 s; *p* value = 0.695), circle cutting (control: 242 s vs. intervention: 220; *p* value = 0.176), or balloon resection (control: 252 s vs. intervention: 204; *p* value = 0.208) (Table 3).

**Table 3:** Comparison of force parameters and task completion time during the final test between in-person and digital training groups (SD = standard deviation, N = Newton, s = seconds)

Outcome variable	In-person (*n* = 21)	Digital (*n* = 19)	*p* value
Overall task completion time in s (mean, SD)	1058.1 (243.6)	877.5 (225.5)	**0.02**
Peg transfer, [mean (SD)]			
Peak force in N	2.66 (1.11)	2.76 (1.22)	0.705
Mean non-zero force in N	0.69 (0.14)	0.68 (0.15)	0.664
Time in s	145.43 (55.77)	134.6 (30.34)	0.695
Circle cutting, [mean (SD)]			
Peak force in N	3.08 (1.228)	2.75 (1.149)	0.44
Mean non-zero force in N	0.75 (0.212)	0.72 (0.309)	0.416
Time in s	241.69 (97.416)	219.66 (109.468)	0.176
Suture and knot, [mean (SD)]			
Peak force in N	4.05 (1.177)	4.42 (1.67)	0.542
Mean non-zero force in N	0.92 (0.183)	0.95 (0.19)	0.684
Time in s	419.38 (132.59)	319.58 (108.737)	**0.015**
Balloon resection, [mean (SD)]			
Peak force in N	6.23 (3.321)	4.76 (2.389)	0.208
Mean non-zero force in N	1.15 (0.391)	0.95 (0.36)	0.078
Time in s	251.61 (127.68)	203.63 (81.363)	0.208

Significant *p* values are highlighted in bold

#### Force during exercise

All students performed the four exercises during the final skill assessment. There were no significant differences in the applied force during the exercises between the control and intervention group. The mean non-zero force showed no significant differences between the groups (Peg: control: 0.69 N vs. intervention: 0.68 N, *p* value = 0.064; circle cutting: control: 0.75 N vs. intervention: 0.72 N, *p* value = 0.416; balloon resection: control: 1.15 N vs. intervention: 0.95 N, *p* value = 0.078; suturing: control: 0.92 N vs. intervention: 0.95 N, *p* value = 0.684) (Table 3).

### Secondary outcomes

Regarding the path length, students in the intervention group had a significantly lower path length for the suturing task compared to the control group (control: 23553 mm vs. intervention: 17555 mm, *p* value = 0.021). In contrast, there was no significant difference for the other exercises. There were also no observable differences for the mean speed of the right hand or left hand.

The volume of motion did not significantly vary between the groups during the four exercises. In contrast, the motion for the left hand during the circle cutting task was significantly lower in the control compared to the intervention group (control: 14735 mm vs. intervention: 19846 mm, *p* value = 0.013). The volume of the left hand was not significantly different during the other exercises.

The assessed depth perception (the distance of the objects used during the exercises) during the balloon resection task was significantly lower in the intervention compared to the control group (control: 17.64 mm vs. intervention: 15.54 mm, *p* value = 0.033). During the other exercises, the depth perception for the right hand did not differ significantly. Similarly, no significant differences for the depth perception of the left hand were observable (Table 4).

**Table 4:** Comparison of secondary outcome parameters during the final test between the in-person and the digital training groups

Outcome parameter	In-person (*n* = 21)	Digital (*n* = 19)	*p* value
Peg transfer, [mean (SD)]			
Total path length in mm	8477.75 (2335.303)	8100.37 (1532.559)	0.735
Mean speed right hand cm/s	3.37 (0.499)	3.35 (0.328)	0.665
Volume of motion right hand in mm	19,375.69 (15,096.519)	15,407.05 (5223.596)	0.35
Depth perception right hand in mm	22.4 (4.249)	20.88 (2.771)	0.409
Mean speed left hand in cm/s	3.18 (0.501)	3.23 (0.327)	0.279
Volume of motion left hand in mm	13,757.84 (6098.3)	13,549.47 (4934.385)	0.735
Depth perception left hand in mm	19.45 (4.198)	18.82 (2.627)	0.818
Circle cutting, [mean (SD)]			
Total path length in mm	9233.3 (2788.437)	8981.97 (4304.92)	0.35
Mean speed right hand cm/s	2.63 (0.602)	2.82 (0.522)	0.303
Volume of motion right hand in mm	16,978.66 (4798.971)	18,795.25 (4626.157)	0.323
Depth perception right hand in mm	19.94 (3.735)	20.33 (3.851)	0.432
Mean speed left hand in cm/s	1.98 (0.263)	1.95 (0.249)	0.745
Volume of motion left hand in mm	14,735,18 (9210,748)	19,845,98 (8073,61)	0,013
Depth perception left hand in mm	18.66 (5.187)	22.05 (7.644)	0.155
Suture and knot, [mean (SD)]			
Total path length in mm	23,552.5 (8379.753)	17,555.37 (5753.367)	**0.021**
Mean speed right hand cm/s	3.14 (0.56)	3.19 (0.418)	0.924
Volume of motion right hand in mm	27,011.93 (11,200.1)	28,760.66 (9935.71)	0.424
Depth perception right hand in mm	27.21 (5.413)	26.13 (4.618)	0.597
Mean speed left hand in cm/s	2.98 (0.514)	2.87 (0.496)	0.393
Volume of motion left hand in mm	25,307.91 (6110.677)	25,203.4 (10,989.434)	0.636
Depth perception left hand in mm	27.64 (3.293)	25,71 (3.782)	0.062
Balloon resection, [mean (SD)]			
Total path length in mm	10,675.87 (6630.286)	8783.61 (3990.212)	0.208
Mean speed right hand cm/s	2.54 (0.534)	2.68 (0.354)	0.176
Volume of motion right hand in mm	14,604.91 (5937.658)	11,855.87 (4599.421)	0.228
Depth perception right hand in mm	17.64 (2,929)	15.54 (2.532)	**0.033**
Mean speed left hand in cm/s	2.36 (0.487)	2.3 (0.425)	0.626
Volume of motion left hand in mm	7787.93 (5105.292)	6501.3 (3851.609)	0.44
Depth perception left hand in mm	13.36 (3.808)	12.91 (2.989)	0.787

Significant *p* values are highlighted in bold

### Assessment of workload during the tasks

**Fig. 4:**
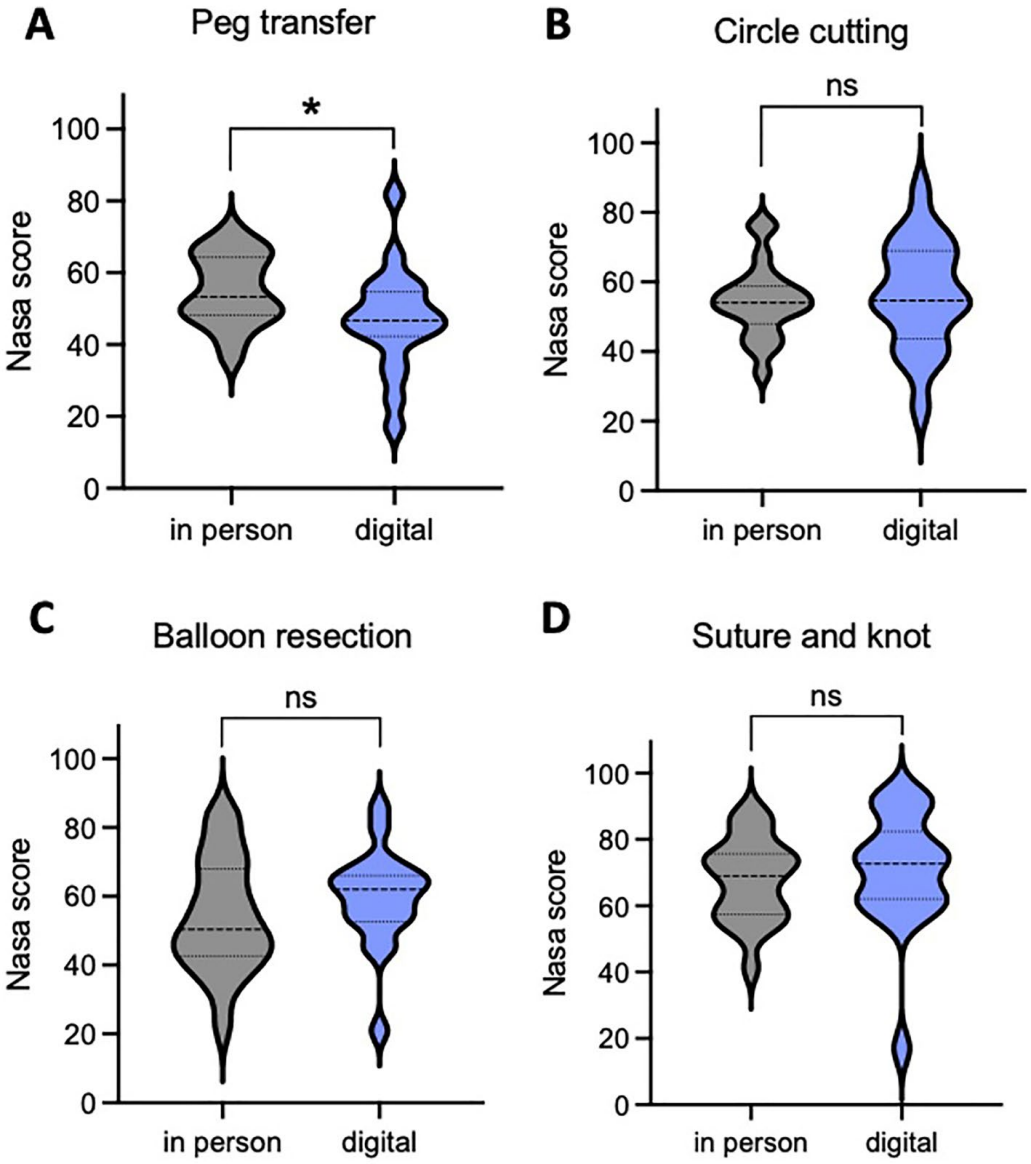
Differences in cognitive workload assessment between the in-person and the digital training groups using the NASA-TLX score. **A** Differences in the NASA-TLX score for the Peg transfer task. **B** Differences in the NASA-TLX score for the circle cutting task. **C** Differences in the NASA-TLX score for the balloon resection task. **D** Differences in the NASA-TLX score for the suture and knot task

The cognitive workload was summarized by the overall NASA-TLX score. While participants of the in-person group showed significantly higher NASA-TLX scores compared to the digital group during the Peg transfer task (control: 55.3 vs. intervention: 47, *p* value = 0.0395), no significant differences between both groups were observed during the other three tasks (circle cutting: control: 54.5 vs. intervention: 56.4, *p* value = 0.6561; balloon resection: control: 54.1 vs. intervention: 59.6, *p* value = 0.2613; and suture: control: 68 vs. intervention: 70.5, *p* value = 0.604) (Fig. 4 and Table 5).

**Table 5:** Comparison of NASA-TLX scores during the final test between the in-person and the digital training groups

Task	In-person (*n* = 21)	Digital (*n* = 19)	*p* value
Peg transfer [mean score (SD)]	55.3 (9.9)	47 (14.3)	**0.0395**
Circle cutting, [mean score (SD)]	54.5 (10.5)	56.4 (15.5)	0.6561
Balloon resection, [mean score (SD)]	54.1 (16.4)	59.6 (13.9)	0.2613
Suture and knot, [mean score (SD)]	68 (12.4)	70.5 (17.7)	0.604

Significant *p* values are highlighted in bold

### Satisfaction with digital and in-person training modalities

The vast majority of students in both groups found their skill improvement to be very good or good (control: 71.4% vs. intervention: 94.7%, *p* value = 0.249). The teaching during the course was rated as very good or good by most participants (control: 90.5% vs. intervention: 94.7%, *p* value = 0.364). In addition, most students found the tutors support to be good or very good (control: 94.3% vs. intervention: 89.5%, *p* value = 0.251).

Participants rated the visibility of the tutor’s instruments as good or very good (control: 81% vs. intervention: 84.2%, *p* value = 0.287) and similarly, the ability to follow the shown instruments (control: 85.8% vs. intervention: 84.2%, *p* value = 0.756). In addition, most students were able to understand the intended instructions by the tutor’s instruments as very good or good (control: 85.7% vs. intervention: 89.4%, *p* value = 0.444).

The use of online teaching in surgical skill acquisition was found to be useful by most of the participants without significant differences between both groups (control: 56.7% vs. intervention: 84.3%, *p* value = 0.096). Students also agreed that simulator training is of importance without significant differences between the groups (control: 100% vs. intervention: 100%, *p* value = 1). Students also agreed that simulator training supports surgeons’ development both in-person (control: 90.4% vs. intervention: 100%, *p* value = 0.353) and online (control: 76.2% vs. intervention: 94.7%, *p* value = 0.248) without significant differences between both groups.

Nearly, all students agreed that simulation training should be part of the surgical training curriculum (control: 95.2% vs. intervention: 100%, *p* value = 1) and would like to have more digital training opportunities to develop their surgical skills (control: 85.7% vs. intervention: 89.4%, *p* value = 0.658). Regarding the choice of training groups, there were significant differences between the groups with the majority of students wanting to be trained in-person (control: 100% vs. intervention: 42.1%, *p* value < 0.001) (Table 6).

**Table 6:** Assessment of the training modality for both groups

Response	In-person (*n* = 21)	Digital (*n* = 19)	*p* value
How would you rate the development of your skills?			0.249
1	3 (14.3)	5 (26.3)	
2	12 (57.1)	13 (68.4)	
3	5 (23.8)	1 (5.3)	
4	1 (4.8)	0 (0)	
5	0 (0)	0 (0)	
How would you rate the teaching during the course?			0.364
1	10 (47.6)	8 (42.1)	
2	9 (42.9)	10 (52.6)	
3	0 (0)	1 (5.3)	
4	0 (0)	0 (0)	
5	2 (9.5)	0 (0)	
How would you rate the ability to follow the instructions of the tutor?			0.756
1	9 (42.9)	6 (31.6)	
2	9 (42.9)	10 (52.6)	
3	1 (4.8)	2 (10.5)	
4	1 (4.8)	1 (5.3)	
5	1 (4.8)	0 (0)	
How would you rate the ability to see the instructor’s instruments?			0.287
1	11 (52.4)	11 (57.9)	
2	6 (28.6)	5 (26.3)	
3	1 (4.8)	1 (5.3)	
4	0 (0)	2 (10.5)	
5	3 (14.3)	0 (0)	
How well did you understand what the instructor’s instruments were showing?			0.444
1	14 (66.7)	10 (52.6)	
2	4 (19)	7 (36.8)	
3	1 (4.8)	2 (10.5)	
4	1 (4.8)	0 (0)	
5	1 (4.8)	0 (0)	
How would you rate the view while performing the exercises?			0.463
1	5 (23.8)	6 (31.6)	
2	6 (28.6)	9 (47.4)	
3	6 (28.6)	2 (10.5)	
4	1 (4.8)	1 (5.3)	
5	3 (14.3)	1 (5.3)	
How would you rate the support by the tutor while performing the exercise?			0.251
1	11 (52.4)	12 (63.2)	
2	9 (42.9)	5 (26.3)	
3	0 (0)	2 (10.5)	
4	0 (0)	0 (0)	
5	1 (4.8)	0 (0)	
Do you think online teaching is useful in teaching surgery skills?			0.096
1	5 (23.8)	12 (63.2)	
2	9 (42.9)	4 (21.1)	
3	5 (23.8)	2 (10.5)	
4	2 (9.5)	1 (5.3)	
5	0 (0)	0 (0)	
Would you rather train in-person or online?			**0.002**
1	1 (4.8)	4 (21.1)	
2	0 (0)	2 (10.5)	
3	0 (0)	6 31.6()	
4	8 (38.1)	4 (21.1)	
5	12 (57.1)	3 (15.8)	
Do you think simulator training is important?			1
1	18 (85.7)	16 (84.2)	
2	3 (14.3)	3 (15.8)	
3	0 (0)	0 (0)	
4	0 (0)	0 (0)	
5	0 (0)	0 (0)	
Do you think simulator training supports residents’ surgical education?			0.353
1	15 (71.4)	16 (84.2)	
2	4 (19)	3 (15.8)	
3	2 (9.5)	0 (0)	
4	0 (0)	0 (0)	
5	0 (0)	0 (0)	
Do you think digital simulator training supports residents’ surgical education?			0.248
1	6 (28.6)	10 (52.6)	
2	10 (47.6)	8 (42.1)	
3	2 (9.5)	0 (0)	
4	3 (14.3)	1 (5.3)	
5	0 (0)	0 (0)	
Do you think simulator training should be part of the general training curriculum?			1
Yes	20 (95.2)	19 (100)	
No	1 (4.8)	0 (0)	
Would you like to be offered more digital surgical training opportunities?			0.658
1	14 (66.7)	15 (78.9)	
2	4 (19)	2 (10.5)	
3	2 (9.5)	2 (10.5)	
4	1 (4.8)	0 (0)	
5	0 (0)	0 (0)	
Which group would you have liked to be trained in?			** < 0.001**
In-person	21 (100)	8 (42.1)	
Digital	0 (0)	11 (57.9)	

Significant *p* values are highlighted in bold

## Discussion

Laparoscopic surgery is a well-established approach and has become the standard of care for some procedures [24]. Inherently, with the increasing complexity of laparoscopic procedures, well-established training modalities are needed to improve laparoscopic education. However, due to limited personnel and financial resources, alternatives to in-person training are needed to extend and optimize training conditions.

In this study, the feasibility of digital training with low-cost surgical simulators was compared to an in-person training approach. It reveals that while there was no difference in overall errors the students who underwent digital training performed the tasks significantly faster than the students of the in-person training group. This underlines the potential of digital training formats for teaching basic laparoscopic surgical skills.

Training outside the hospital has been the topic of recent research [25]. Studies have shown that digital training is feasible for skill development. A study by Ramadan et al. demonstrated that participants self-assessed their skills significantly better after an online education program [26], while studies by Joosten et al. and Falcioni et al. have shown digital and unsupervised training to be effective in developing surgeons’ laparoscopic skills [13, 27]. The study by Joosten et al. assessed participants self-evaluated skill development, which increased significantly during the use of the simulator at home [13].

Most recently, Falcioni et al. compared the digital and in-person setting for teaching pediatric laparoscopic surgical skills. There were no differences in performance between the two groups at the end of the study [28]. This falls in line with our study results, which also showed no overall significant differences in errors between the two groups. Unlike their study, in addition to assessing each task individually, this study also statistically compared the overall time needed to complete all tasks. Here, the in-person training group required about 20% more time than the digital group. When assessing the study by Falcioni et al. a similar difference of about 12% between the two groups in favor of the telementoring group becomes apparent; however, this was not statistically assessed. This similarity underscores the results of this study, indicating that digital/telementoring leads to faster completion of the tasks.

Beside comparable skills at the final test, we also questioned students about the two training programs. Our results align with previous studies showing that at-home training satisfies trainees and is a feasible training set-up [26]. Nevertheless, students in the digital group would have liked to be trained in-person. There are many possible reasons for that and a combination of initial in-person and subsequent digital training might be a suitable adjustment. Joosten et al. showed that participants in their study would prefer that online training should be performed after initial in-person training [29]. It is very likely that a combination of both modalities is possible as well, thereby satisfying both preferences.

The study by Thinggaard et al. investigated the outcomes of additional at-home training opportunities on students’ time to finish a laparoscopic training program. Even if the students did not show significant skill differences, the ability to train at home was well perceived and resulted in more frequent training, allowing a more personal approach to training [30]. The study showed that students were able to choose their own training schedule when training at home without supervision. Nevertheless, students complained about the missing feedback [12]. Our training provided supervisor feedback while training at home and was well perceived by students. Therefore, future studies should also evaluate the best implementation of un- and supervised digital training.

In addition, students valued the personal environment and reduced distractions while training at home [12]. Training at home might reduce distractions, such as dehydration and noise, known to interfere with surgical novices’ performance [23]. Additionally, the assessed cognitive workload showed no clear superiority of one training environment, underlining the comparability of both training approaches.

Using low-cost trainers, we further wanted to show the usability of a low-cost personalized environment and its feasibility for skill development. The usability of low-cost portable simulators and their compatibility with more expensive simulators has been shown previously [10]. Similar to our study, a study by van der Ar et al. also showed a high likability of low-cost trainers by residents [31].

Nevertheless, digital training also has its pitfalls. The ability of at-home training might further diminish the separation of work and off-time [25]. Such work-life overlap and extension of already long working hours is associated with an increased risk of burnout and needs to be evaluated carefully [32].

## Conclusion

This study shows that our basic laparoscopic curriculum is applicable to a digital training environment. Digital training proved to be equal to in-person training in skill development and student perception and students performed the tasks faster in the final assessment. Nevertheless, further studies need to evaluate the best suitable combination of in-person and digital training, as well as the potential resource and time savings. Even with such advancements, the problem of insufficient training time still exists. Surgical skill acquisition is a definitive part of residents training and therefore, it is especially important to not shift training to the off-work time. Apart from these caveats, digital training has the ability to personalize laparoscopic training and increase its accessibility, including the accessibility to low-income countries.

## Limitations

This study was performed on medical students without any prior experience in laparoscopic surgery. These surgical novices might lack the knowledge of standard surgical curricular and conclusions of their subjective assessment should be evaluated in that context. Additionally, the ratio of trainers to trainees was rather low. Especially in the unknown digital format this might have affected the training. Further research and adjustment of digital training formats is needed. While students in the digital group were advised to not train between sessions, due to privacy and logistical reasons this was not able to be monitored between sessions. Therefore, students might have performed additional training. Even if this would be beneficial in a real-world setting for surgical skill development it might have affected students’ skills at the final evaluation. The design of the study does not allow for the in-person comparison of the two training regimens, limiting the evaluation of students’ notes on the preference of either training option.

## Supplementary Information

Below is the link to the electronic supplementary material.

Supplementary file1 (DOCX 14 KB)

## Data Availability

The data are available upon reasonable request to the corresponding author.
