# Correction: Comparison of distance versus in-person laparoscopy training using a low-cost laparoscopy simulator—a randomized controlled multi-center trial

**DOI:** 10.1007/s00464-024-11399-1

**Published:** 2024-11-05

**Authors:** Mark Enrik Geissler, Jean-Paul Bereuter, Rona Berit Geissler, Guus Mattheus Johannes Bökkerink, Luisa Egen, Karl-Friedrich Kowalewski, Caelan Haney

**Affiliations:** 1 Else Kroener Fresenius Center for Digital Health, Faculty of Medicine and University Hospital Carl Gustav Carus, TUD Dresden University of Technology, 01307 Dresden, Germany; 2 Department of Urology and Urosurgery, University Medical Centre Mannheim, University of Heidelberg, Mannheim, Germany; 3 Princess Máxima Center for Pediatric Oncology, Princess Maxima Center, Utrecht, The Netherlands; 4 Division Intelligent Systems and Robotics in Urology, German Cancer Research Center (DKFZ), Heidelberg, Germany; 5 DKFZ Hector Cancer Institute at the University Medical Center, Mannheim, Germany

**Correction to: Surgical Endoscopy (2024) 38:6527–6540** 10.1007/s00464-024-11069-2

The original online version of this article was revised to correct the presentation of the name of corresponding author Mark Enrik Geissler.

The original article has been corrected.

