# Supplementary material for: Comparison of distance versus in-person laparoscopy training using a low-cost laparoscopy simulator—a randomized controlled multi-center trial
Source: Surg Endosc. 2024 Sep 13;38(11):6527–40. doi: 10.1007/s00464-024-11069-2 (PMC11525308; doi:10.1007/s00464-024-11069-2)
Supplement: Supplementary file 1 — Supplementary file1 (DOCX 14 KB) [file 464_2024_11069_MOESM1_ESM.docx]

| **Error Definitions** | | |
| --- | --- | --- |
| Task | Points | Definition |
| **Peg-transfer task** | 0 | No triangle droped |
|  | 1 | Per triangle droped |
| **Circle-cutting task** | 0 | 0 - 5 mm cut out of circle |
|  | 1 | For each cut > 5 - 10 mm out of circle |
|  | 2 | For each cut > 10 mm out of circle |
| **Resection task** | 0 | No perforation of balloon |
|  | 1 | Micro perforation, water is only leaking of pressure is applied |
|  | 2 | Macro perforation, water leaks without applied pressure |
| **Suture and knot tying task** |  |  |
| Suture precision | 0 | Suture through both dots |
|  | 1 | Suture through one dot |
|  | 2 | Suture through no dot |
| Suture closeness | 0 | Sides of the penrose are attached |
|  | 1 | Sides of the penrose are close, but do not touch |
|  | 2 | Sides of the penrose are not closer |
| Knot tightness | 0 | Knot tight, withstands manipulation |
|  | 1 | Knot loosens with manipulation |
|  | 2 | Knot visually loose |

**Supplementary Table 1:** Assessment of Errors during task performance. Adapted after Bechtolshiem et al. [(19)](https://www.zotero.org/google-docs/?azBWJY).
